# Profiling of koumiss microbiota and organic acids and their effects on koumiss taste

**DOI:** 10.1186/s12866-020-01773-z

**Published:** 2020-04-10

**Authors:** Hai Tang, Huimin Ma, Qiangchuan Hou, Weicheng Li, Haiyan Xu, Wenjun Liu, Zhihong Sun, Halatu Haobisi, Bilige Menghe

**Affiliations:** 1 Key Laboratory of Dairy Biotechnology and Engineering, Ministry of Education, Inner Mongolia Agricultural University, Hohhot, 010018 People’s Republic of China; 2 Key Laboratory of Dairy Products Processing, Ministry of Agriculture, Inner Mongolia Agricultural University, Hohhot, 010018 People’s Republic of China; 3 Inner Mongolia International Mongolian Hospital, Hohhot, 010018 People’s Republic of China

**Keywords:** Bacterial diversity, Yeast diversity, Organic acids, Taste, Koumiss

## Abstract

**Background:**

Koumiss is a naturally fermented mare’s milk. Over recent decades, numerous studies have revealed the diversity of lactic acid bacteria in koumiss. However, there is limited information available regarding its secondary major component yeast profile.

**Results:**

A total of 119 bacterial and 36 yeast species were identified among the 14 koumiss samples. The dominant bacterial species in koumiss were *Lactobacillus helveticus*, *Lactobacillus kefiranofaciens*, *Lactococcus lactis*, *Lactococcus raffinolactis*, and *Citrobacter freundii.* The main yeast species were *Dekkera anomala*, *Kazachstania unispora*, *Meyerozyma caribbica*, *Pichia sp.BZ159*, *Kluyveromyces marxianus*, and uncultured *Guehomyces*. The bacterial and yeast Shannon diversity of the Xilinhaote-urban group were higher than those of the Xilingol-rural group. The most dominant organic acids were lactic, acetic, tartaric, and malic acids. Lactic acid bacteria species were mostly responsible for the accumulation of those organic acids, although *Kazachstania unispora*, *Dekkera anomala*, and *Meyerozyma caribbica* may also have contributed. Redundancy analysis suggested that both bacteria and yeast respond to koumiss flavor, such as *Lactobacillus helveticus* and *Dekkera anomala* are associated with sourness, astringency, bitterness, and aftertaste, whereas *Lactococcus lactis* and *Kazachstania unispora* are associated with umami.

**Conclusions:**

Our results suggest that differences were observed in koumiss microbiota of Xilinhaote-urban and Xilingol-rural samples. The biodiversity of the former was higher than the latter group. Positive or negative correlations between bacteria and yeast species and taste also were found.

## Background

Koumiss is a naturally fermented mare’s milk with a long history of consumption in the Mongolian steppes. Its name is derived from the Kumanese, who survived as a Kumane River tribe on the central Asian steppes until 1235. When fully renneted, the proteins in horse milk form no visible curd [1], and it is ivory or slightly yellow in color with a sharp acidic and alcoholic flavor [2]. The flavor characteristics of koumiss were recorded in the Mongolian medical book as follows: “milk taste sour, slight sweet, a bit acerbity”. Koumiss has been used in the treatment of some diseases in Mongolian traditional medicine [3]. Koumiss is a typical yeast-lactic fermented product that is made with specific strains of lactic acid bacteria (LAB) and yeast [4]. The Lactobacillus strains isolated from koumiss show good performance as probiotics [5]. Over recent decades, numerous studies using culture-dependent and -independent methods have revealed the diversity of LAB in koumiss [6–8]. However, there is limited information available regarding its yeast profile [9, 10] or sensory characteristics.

The flavor of dairy products is directly and indirectly derived from factors such as the raw milk quality, production process, and activities of diverse microbial populations [11]. In particular, the rich bacterial and yeast microbiota of yeast-lactic fermented milk are responsible for the unique flavor properties [12]. Usually, biodiversity of naturally fermented milk is varied with sample types and geographical origins [8, 13]. Moreover, koumiss produced by herdsmen is particularly sour; this type of koumiss was thought to have the most medicinal value, but its taste is unacceptable to some consumers. Therefore, we collected samples from the city (Xilinhaote-urban group) and the surrounding rural area (Xilingol-rural group) to study the differences in koumiss microbiota.

During milk fermentation, the concentrations of some organic acids (lactic and acetic acid) increase, while those of other organic acids (citric acid, etc.) decrease [14]. Short-chain carboxylic acids are one of the most important differential contributors to the flavor of milk products. These acids are derived from lipolysis, carbohydrate metabolism, or amino acid metabolism [15, 16]. We assessed the organic acid content of koumiss.

Chemical composition analysis provides quantitative data that are not instructive in terms of analysis of taste; therefore, we employed a taste-sensing system with advanced taste sensors that were developed based on correlations with human sensory scores, and are applied in assessments of milk and beverage products [17–20]. Traditional fermented foods may be significantly improved by using starter cultures selected based on multiple considerations [21]. The present study assessed the correlations among organic acid content, taste, and koumiss microbiota to provide data to guide the development of a koumiss starter culture, with improved and controlled fermentation, for commercial use.

## Results

### Koumiss bacteria and yeast: composition and diversity

**Fig. 1:**
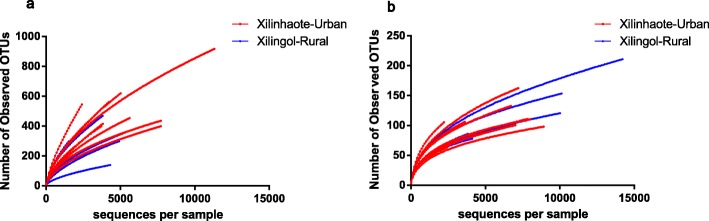
Rarefaction curve of the bacterial (**a**) and yeast (**b**) diversity

**Fig. 2:**
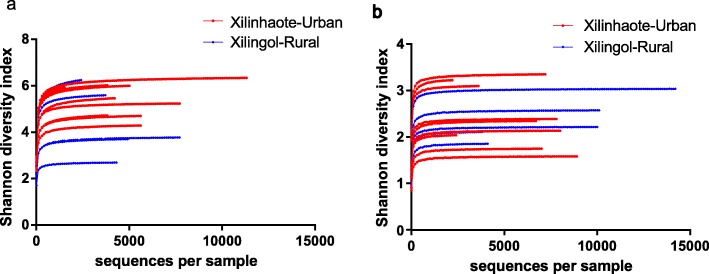
Shannon diversity index of bacterial (**a**) and yeast (**b**) composition

Sequencing of the full-length 16S rRNA gene using the SMRT technology revealed high taxonomical resolution of the Xilinhaote-urban and Xilingol-rural groups at the species level (Fig. 1). The bacterial Shannon diversity index of the Xilinhaote-urban group was slightly higher than that of the Xilingol-rural group (*p* = 0.068), whereas the yeast Shannon diversity index was higher in Xilinhaote-urban than Xilingol-rural samples (*p* = 0.001) (Fig. 2). The most diverse bacterial species was *Lactobacillus lactis* (*L. lactis*); it had a lower abundance in Xilingol-rural samples than Xilinhaote-urban samples (*p* = 0.012). There were no significant differences in dominant yeast species abundance.

**Fig. 3:**
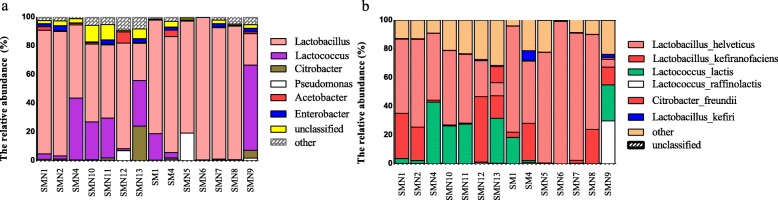
The relative abundance of bacterial genus (**a**) and species (**b**)

**Fig. 4:**
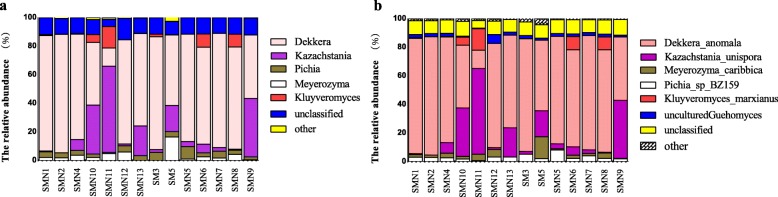
The relative abundance of fungal genus (**a**) and species (**b**)

A total of 119 bacterial and 36 yeast species were identified among the 14 koumiss samples. The bacterial and yeast species with a relative abundance of > 1% are summarized (Tables 1 and 2) and presented (Figs. 3 and 4). The homotypic synonym of *Kazachstania unispora* (*K. unispora*) is *Saccharomyces unisporus.* Yeast species with fluctuations in abundance of < 5% include *Meyerozyma caribbica* (*M. caribbica*), *Pichia sp.BZ159* (*P. BZ159*), and uncultured *Guehomyces.*

**Table 1:** Average relative abundance of bacteria in different taxa

Phylum	average abundance (%) ± SE	Genus	average abundance (%) ± SE	Species	average abundance (%) ± SE
Firmicutes	86.4 ± 10.8	Lactobacillus	69.5 ± 24.6	Lactobacillus helveticus	53.3 ± 27.5
				Lactobacillus kefiranofaciens	13.4 ± 14.6
				Lactobacillus kefiri	0.8 ± 1.9
		Lactococcus	15.6 ± 19.2	Lactococcus lactis	12.8 ± 15.1
				Lactococcus raffinolactis	2.3 ± 7.9
Proteobacteria	13.6 ± 10.7	Pseudomonas	2.3 ± 5.2	–	–
		Acetobacter	1.5 ± 2.12	–	–
		Enterobacter	1.4 ± 1.2	–	–
		Citrobacter	2.5 ± 6.4	Citrobacter freundii	1.1 ± 3
unclassified	0	unclassified	3.6 ± 3.6	unclassified	11.5 ± 7.3

**Table 2:** Average relative abundance of yeast in different taxa

Phylum	average abundance (%) ± SE	Genus	average abundance (%) ± SE	Species	average abundance (%) ± SE
Ascomycota	97.5 ± 1.3	Dekkera	64.2 ± 19.8	Dekkera anomala	64.2 ± 19.8
		Kazachstania	14.2 ± 18.7	Kazachstania unispora	14.1 ± 18.6
		Meyerozyma	3 ± 4.8	Meyerozyma caribbica	3 ± 3.9
		Pichia	3.7 ± 1.8	Pichia sp.BZ159	3 ± 1.8
		Kluyveromyces	3.3 ± 4.2	Kluyveromyces marxianus	2.8 ± 4.9
Basidiomycota	2.4 ± 1.3	unclassified		unculturedGuehomyces	2 ± 1.3
unclassified	0.1 ± 0.1	unclassified	10.9 ± 2.1	unclassified	9.3 ± 1.3

These tables didn’t show the species relative abundance was lower than 1%

### Organic acids in koumiss

**Fig. 5:**
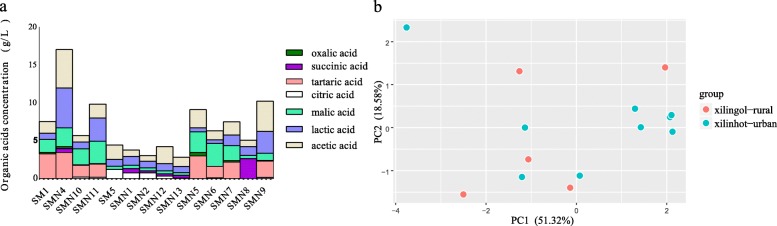
Koumiss organic acids profiling and PCA analysis

The organic acids detected in koumiss included oxalic, tartaric, malic, lactic, acetic, citric, and succinic acid, with an average content of 6.54 g/L. The most dominant acids across all samples were lactic (1.42 g/L, 21.7%), acetic (1.78 g/L, 27.3%), tartaric (1.26 g/L, 19.2%), and malic (1.39 g/L, 21.2%) acids (Fig. 5a). The mean concentration of citric acid was higher in Xilinhaote-urban samples (0.39 g/L) than Xilingol-rural samples (0.06 g/L, *p* < 0.05).

All major organic acids were analyzed by principal component analysis. The first two components explained 69.90% of the variance. PC1 accounted for 51.32% of the variance and well-explained the separation of the Xilinhaote-urban and Xilingol-rural samples. Xilinhaote-urban samples formed a tighter cluster than the Xilingol-rural samples, which were more diffuse along PC1 (Fig. 5b).

### The potential symbiotic relationship between yeasts and LAB

**Fig. 6:**
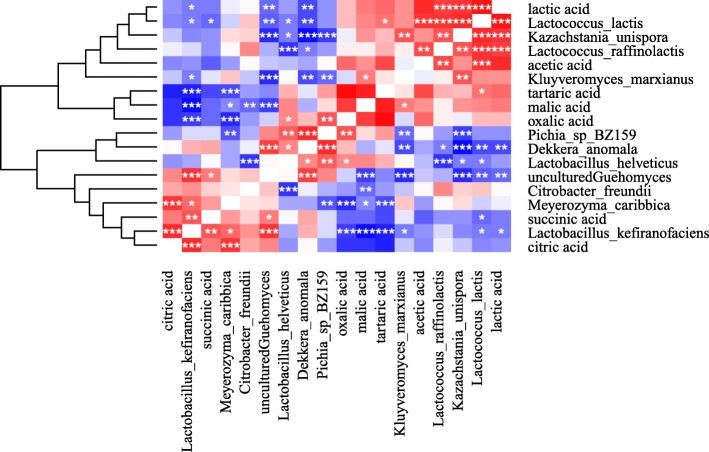
The Correlation of Koumiss Microbiota and Organic Acids. There are no shown the relationship between the organic acids. In the figure “*” represents *p* < 0.05, “**” represents *p* < 0.01,“***” represents *p* < 0.001

In addition to the associations of bacterial and yeast species compositions, a correlation between the abundance of yeast species and LAB was also observed (Fig. 6). The abundance of the predominant bacterial species *Lactobacillus helveticus* (*L. helveticus*) was positively correlated with that of *Dekkera anomala* (*D. anomala*) and *P. BZ159* (*p* < 0.01), and negatively correlated to that of *K. unispora* (*p* < 0.05)*.* The abundance of the second most dominant species, *L. lactis*, was positively correlated with that of *K. unispora* (*p* < 0.01) and negatively correlated with that of *D. anomala* and *uncultured Guehomyces* (both *p* < 0.05)*.*

### Correlations among koumiss microbiota, organic acids, and taste

A complex correlation between koumiss microbiota and organic acid accumulation was observed (Fig. 6). Lactic acid accumulation was positively correlated with the abundance of *Lactococcus raffinolactis* (*L. raffinolactis*)*, L. lactis*, and *K. unispora*, and negatively correlated with that of *Lactobacillus kefiranofaciens* (*L. kefiranofaciens*)*, D. anomala*, and *uncultured Guehomyces*. Interestingly, the abundance of *L. raffinolactis* and *L. lactis* was positively correlated with acetic acid accumulation*.* The abundance of *L. kefiranofaciens* and *M. caribbica* was positively correlated with citric acid accumulation, and negatively correlated with tartaric and malic acid accumulation. The abundance of *L. kefiranofaciens* and *unculturedGuehomyces* was positively correlated with that of succinic acid, whereas *L. lactis* abundance had a negative correlation.

In the grouping study, Xilingol-rural samples exhibited stronger sourness (*p* = 0.06) and aftertaste-A (astringent aftertaste, *p* = 0.04) than Xilinhaote-urban samples (Figure S1), but there were no differences in any other taste parameter.

Sourness, astringency and aftertaste-A, bitterness and aftertaste-B, and richness clustered together. Moreover, microbes making a positive contribution to sourness-related tastes, including *L. helveticus*, *L. kefiranofaciens*, *D. anomala*, *P. BZ159*, uncultured *Guehomyces,* were negatively correlated with sweetness, umami and saltness.

**Fig. 7:**
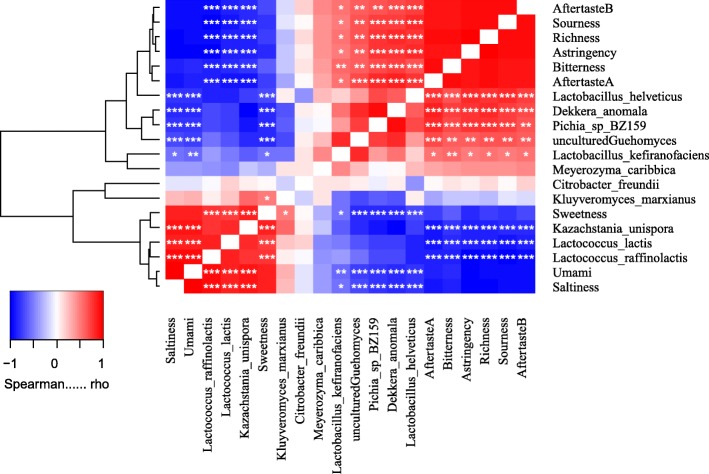
The correlation of koumiss microbiota and the taste. There are no shown the relationships between the species each other and taste in the figure. In the figure “*“represents *p* < 0.05, “**” represents *p* < 0.01,“***” represents *p* < 0.001

Saltiness, umami, and sweetness were clustered together, and were positively correlated with *L. lactis*, *L. raffinolactis*, and *K. unispora*; these species were negatively correlated with sourness-related tastes (Fig. 7)*.* Only *Kluyveromyces marxianus* (*K. marxianus*) was positively correlated with sweetness (*p* < 0.01).

**Fig. 8:**
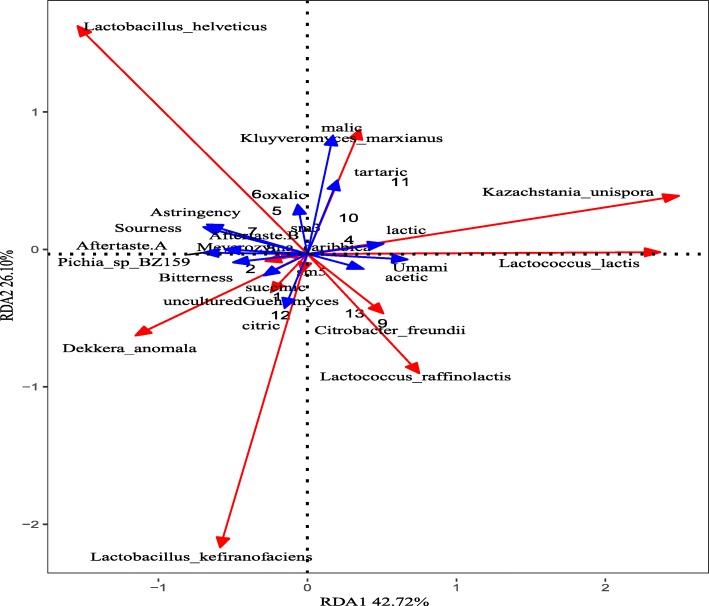
Redundancy analysis of koumiss Microbiota on organic acids and taste. The numbers stands for samples, exception sample ‘sm3’and‘sm5’. The blue arrows represent the response variables (organic acids and taste), and the red arrows represent the species variables

Redundancy analysis (RDA) was performed to identify the species in koumiss associated with the various types of tastes and organic acids (Fig. 8). *L. helveticus* and *D. anomala* were strongly associated with sourness, astringency, bitterness, and aftertaste. *L. lactis and K. unispora* were strongly associated with umami and lactic and acetic acids. *K. marxianus* was associated with malic, tartaric, and oxalic acids. *L. kefiranofaciens* was associated with citric acid.

## Discussion

### Bacterial and yeast diversity

Koumiss taste can be classified into three types according to the lactic acid content: strong (0.91–1.08%), moderate (0.73–0.90%), and weak (0.54–0.72%) [22]. According to this classification, the koumiss in the present study was classified as a weak form from fermented mare’s milk.

Many studies on koumiss bacterial or yeast diversity used culture- or molecular biology-based methods; however, results varied significantly due to differences in the analysis techniques applied, research approaches, and geographic regions [23–26]. In the current study, we applied long-read SMRT sequencing technology (Pac Bio) with high-resolution phylogenetic microbial community profiling [27] to identify a novel dominant bacterial species*, Citrobacter freundii* that had not been reported previously; the dominant yeast species were *M. caribbica* and uncultured *Guehomyces.* One observation in our study that was inconsistent with previous research was that the abundance of *Enterobacter* genus was < 1%; this may be because the samples were freshly fermented koumiss, whereas *Enterobacter* was found to be enriched during later fermentation and is related to flavor ripening [28]. In our study, the abundance of *Acetobacter* genus was lower (1.5 ± 2.13%), and mainly consisted of *Acetobacter malorum*, *Acetobacter pasteurianus*, and *Acetobacter orientalis*. The heterofermentative species included a small amount of *Lactobacillus kefiri* (0.82 ± 1.86%) and *Leuconostoc mesenteroides* (0.53 ± 1.45%).

Regarding most diverse bacterial composition between two groups, the mesophilic bacteria *L. lactis* was absent from Xilingol-rural koumiss samples, there may be a competition relationship between *L. lactis* and *L. helveticus*, because *L. helveticus* utilizes galactose moieties and glucose, whereas *L. lactis* only utilizes the latter [29]. Regarding interactions among bacterial species, in a previous study, mixed cultures of *Lactococcus lactis ssp. lactis* and *L. raffinolactis* stimulated more acid production during skim milk fermentation [30]. One possible reason for this is that *L. raffinolactis* strains are able to ferment α-galactosides [31], which are not utilized by *L. lactis* strains. However, in the current study, although *L. lactis* and *L. raffinolactis* showed a trend toward a positive correlation in their abundances, it was not statistically significant. In contrast, the abundances of *L. kefiranofaciens* and *L. raffinolactis* were positively correlated. *L. kefiranofaciens* is rarely isolated from koumiss [32] but is a key LAB species in kefir grain ecosystems [33]. *Citrobacter freundii* (*C. freundii*) is a common component of the gut microbiome of healthy humans, and of soil, and is a widely distributed opportunistic pathogen [34]. *C. freundii* was found in one sample at an abundance of 11.37% but was present in very small amounts in other samples; thus, the high-abundance sample may have been due to contamination.

Yeasts producing koumiss (alcoholic) can ferment lactose or galactose. In the current study, the lactose-fermenting yeasts were *D. anomala* and *K. marxianus*, while the galactose-fermenting yeasts included *K. unispora* and *M. caribbica*.

The predominant yeast species, *D. anomala,* belongs to the genera *Dekkera* (Brettanomyces), which is known for its ability to spoil alcoholic beverages by producing acetic acid and unpleasant flavors and odors [35, 36]. *D. anomala* is the teleomorph of *Brettanomyces anomalus*; it differs morphologically and physiologically from the other species in the genus *Dekkera* in terms of the formation of blastese and the ability to ferment lactose [37]. *D. anomala IGC 5153* exhibited limited ability to use weak acids as its only source of carbon and energy. The acetate carrier was not only inducible, but also subject to glucose repression, similar to the carriers identified in acetic acid–grown cells of yeasts such as *S. cerevisiae*. *D. anomala* grew in acetic acid-containing medium with a relatively high total acid content, and over a broad pH range including pH 3.5. *D. anomala* isolates were obtained from Inner Mongolia Koumiss samples and shown by polymerase chain reaction denaturing gradient gel electrophoresis (PCR-DGGE) to be present in an amount of 7.29% [9]; this differs from our sequencing results, which showed amounts of 13–83%. In other milk products, *D. anomala* was isolated from kefir, koumiss, and Shubat [25, 32, 38]. Feta cheese made from pasteurized ewe’s milk contains *D. anomala*. Because wood is an ecological niche for this strongly fermented species, its presence is likely attributable to the wooden tables used for dry salting the cheese blocks [39]. In the current study, the two samples with the lowest abundance of *D. anomala* were fermented in crock using a traditional method. This suggests that non-wood containers and stirrers could be used to restrain the overgrowth of this species. In our koumiss samples, *D. anomala* may have partially contributed to the production of acetic acid, but its correlation with the acetic acid content was negative and nonsignificant (Fig. 6). This may be because it can transport acetic acid into cells as a carbon source to produce CO_2_; this is known as the Crabtree effect and underlies the “make-accumulate-consume” life strategy of organisms such as *S. cerevisiae* [40, 41]. It is a strongly fermented species that may bloom at the beginning stage of fermentation.

The second most common yeast species was *K. unisporus*. A previous study found that its dominance increased at higher altitudes [42], but in koumiss samples from the Xilingol prairie, where latitude does not vary greatly, the abundance varied widely from 0.03 to 60%. *K. unisporus* has been found in various dairy products. It is a galactose-fermenting yeast species and the principal microorganism responsible for alcoholic fermentation, but is a slow producer of ethanol and performs a clean fermentation in milk and whey. Thiamine is the only vitamin required exogenously for the growth of *K. unisporus*; therefore, thiamine may be a key factor determining its prevalence [43]. These samples are weakly fermented koumiss, there is a limited amount of galactose in the system, which may also be another factor.

The yeast Shannon diversity index of Xilinhaote-urban samples was significantly higher than that of Xilingol-rural samples, mainly because of the absence of *K. marxianus* in most Xilingol-rural samples. *K. marxianus* utilizes lactose to excrete some glucose and galactose into milk and weakly metabolizes citrate and succinic acids to produce ethanol, glycerol, acetic acid, and propionic acid [10]. *K. marxianus* is also emerging as a new platform for the production of flavor and fragrance compounds [44]; it is used for the production of phenylethyl alcohol, responsible for the aroma of roses in cheese whey [45].

*M. caribbica*, *P. BZ159*, and uncultured *Guehomyces* were detected in the koumiss samples, and their presence may be attributable to the non-starter yeast species. The species within the genus Pichia are methylotrophic yeasts [46]. *P. BZ 159* has two alcohol oxidase genes, *aox* A and *aox* B, which can oxidize alcohols to aldehydes with concomitant production of hydrogen peroxide. Glucose strongly suppresses the expression of both genes [47].

### Koumiss organic acid profiling

For the first time, we systematically identified the organic acids responsible for the sour taste of koumiss. The organic acid composition did not directly reflect the grouping of samples; in fact, it better-reflected the fermentation process. Most organic acids produced by microbes can be divided into two main groups depending on their metabolic origin and the main metabolite sequence of the aerobic organism: those associated with the tricarboxylic acid cycle (TCA) and glycolysis arising from the oxidation of glucose; and those using glucose to produce organic acids. Citric, lactic, and malic acids fall into the first group, whereas acetic acid should be considered a biotransformation of ethanol. Succinic acid is mostly produced using chemical methods. *K. unisporus* ferments certain monosaccharides to produce succinic acid and acetic acid during ethanol fermentation [48], where the lactic and acetic acid content is the main indicator of the fermentation dynamic. In the present study, the average acetic acid content was higher than the lactic acid content, which may be the result of weak fermentation; as the fermentation proceeds, lactic acid may become dominant over acetic acid. In the bacterial community, most bacteria are homofermentative species and the less-abundant heterofermentative species, including *L. kefiri* and *Leuconostoc mesenteroides*, produce acetic acid in koumiss*.* The trace amounts of acetic acid may by generated from ethanol by acetic acid bacteria [49]. More importantly, few LAB species are able to co-metabolize citrate with fermentable sugars to produce acetic acid. Specifically, variants of *L. lactis* (i.e., *L. lactis* subsp.lactis biovar diacetylactis) are capable of citrate utilization because they possess a plasmid-encoded citrate transporter gene [50]. Our samples may have contained variants of *L. lactis*. In the yeast community, acetic acid-producing species include *D. anomala* and *K. unisporus*.

There were significant lower content of citric acid in Xilingol-rural than Xilinhaote-urban groups. Citric acid is a common constituent of milk; its abundance in mare’s milk is < 2.3 g/kg. Moreover, it is a substrate for certain LAB, which use it to form aromatic substances such as acetoin and diacetyl [51, 52]. In our study, the more sour Xilingol-rural samples had lower citric acid content, which was consistent with the result that its content decreased with fermentation. During fermentation, the citric acid content did not remain steady. First, microbes use glucose to produce citric aacid via the TCA cycle, and then to form aromatic precursors. In addition, citric, malic, and succinic acids are components of the TCA, which together contributed to sourness and bitterness. There are subtle differences in taste: malic acid has a tart, smooth, and long-lasting flavor [48], whereas citric acid has a sour and bitter aftertaste that lasts only for a short time. Succinic acid has an acidic and salty/bitter taste and is savory in cheese [53].

Succinic acid is a good indicator of yeast inoculation status. It is the main organic acid produced by yeast and is formed in the glyoxylate cycle via oxidation of isocitrate, as well as in the reductive citric acid cycle [54]. In the current study, succinic acid accumulation correlated with the abundance of uncultured *Guehomyces* and *L. kefiranofaciens*.

### Koumiss microbiota: association with taste

In the past, more attention was paid to bacterial as opposed to yeast species, even though both bacteria and yeast contribute to unique koumiss fermentation. The presence of yeast in dairy products contributes to flavor formation via the synthesis of a variety of chemical compounds [55]. The koumiss starter culture only containing a few strains of microbes [56] may loss many benefits of koumiss consumption. In tradition, a small aliquot of koumiss from end product is retained for use as starters for the next batch, this method was out of accurate control of fermentation. Redundancy and correlation analysis suggest that those species are associated with tastes and organic acids in koumiss. To control excessive sourness, astringency, and bitterness during koumiss production, our data suggest that the amounts of the post-acidifying strains *L. helveticus* and *D. anomala* should be reduced, or those of *L. lactis* and *K. unispora* increased, in the koumiss starter culture.

Studies of the interaction between bacteria and yeast in fermented foods yield inconsistent results due to differences in strains, substrates and food matrices [26, 55, 57, 58]. During the fermentation of food products, five types of interactions occur between, and may play a role in the consortia of, microbes [59]. However, the putative correlations identified could not be distinguished in terms of whether they were direct or indirect, nor could directionality be established [60]. A study of the interaction between *L. lactis* and yeast found that *L. lactis* produced acetic acid when co-cultured with some yeasts; moreover, the growth of *L. lactis* was enhanced in most co-cultures in UHT milk [61]. The present study observed a potential symbiotic relationship between bacteria and yeast; however, the mechanism underlying this putative relationship is unclear so further research is necessary.

## Conclusions

The dominant microbes in the weak form of koumiss derived from fermented mare’s milk (in the Xilingol region) analyzed in this study includes *L. helveticus*, *L. kefiranofaciens*, *L. lactis*, *L. raffinolactis, D. anomala*, *K. unispora*, *M. caribbica*, *P. BZ159*, *K. marxianus* and *uncultured Guehomyces*. The abundance of *L. lactis* significantly lower in Xilingol-rural samples than Xilinhaote-urban samples. Lactic, acetic, tartaric, and malic acids were the main organic acids that accumulated during koumiss fermentation. LAB species were mostly responsible for the accumulation of those organic acids, although *K. unispora*, *D. anomala*, and *M. caribbica* may also have contributed. The RDA showed that *L. helveticus* and *D. anomala* are associated with sourness, astringency, bitterness, and aftertaste, whereas *L. lactis* and *K. unispora* are associated with umami. The interaction between bacteria and yeast could be demonstrated more convincingly an orthogonal single-strain fermentation experiment combined with a meta-transcriptome study. Our study provides reference values to improve understanding of the interaction between bacteria and yeast in koumiss. These results could inform the selection of multiple-strain cultures for use as bacteria and yeast mixed starter cultures.

## Methods

### Sample collection

Fourteen fresh naturally fermented koumiss samples were collected from the Xilingol region of Inner Mongolia and stored at − 80 °C until analysis. The samples were classified into two groups according to the collection site. “Xilinhaote-urban” samples were collected around Xilinhaote city, where koumiss is produced at the ranch scale (including SMN1, SMN2, SMN4, SMN11, SMN12, SMN13, SM3 alternative to SM1, SM5 alternative to SM4), whereas “Xilingol-rural” samples were collected from herdsmen located 100–150 km from Xilinhaote city (including SMN5, SMN6, SMN7, SMN8, and SMN9). We evaluated the koumiss quality and sensory by two senior dairy researchers in situ when samples were collected.

### DNA extraction and PCR amplification

Genomic DNA was extracted using an Omega DNA Soil Kit (Omega Bio-Tek, Inc., Norcross, GA, USA) following the manufacturer’s instructions. The quality of the extracted DNA was verified using 1.0% agarose gel electrophoresis and a NanoDrop ND-1000 spectrophotometer (Thermo Fisher Scientific, Waltham, MA, USA). All extracted DNA samples were stored at −20°C until analysis.The full-length sequences of 16S rRNA from genomic DNA were amplified using barcoded universal primers 27F and 1492R. Despite the wide use of ITS rRNA as a fungal DNA barcode, its ability to resolve relationships at higher taxonomic levels is inferior to that of many protein-coding genes. Therefore, we combined ITS with the large rRNA subunit (28S rRNA) to differentiate target organisms from close phylogenetic neighbors [62, 63]. The 28S rRNA and ITS genes of yeast were amplified using ITS/28S barcoded primers (forward, 5′-TCCGTAGGTGAACCTGCGG-3′; reverse 5′-TCCTGAGGGAAACTTCG-3′). The PCR program was as follows: 95 °C for 2 min, 98 °C for 20 s, 60 °C for 15 s, 72 °C for 45 s, and a final extension of 72 °C for 90 s. The PCR was performed over 28 cycles for bacteria and 30 cycles for yeast. HiFi HotStart ReadyMix PCR Kit (Kapa Biosystems, Wilmington, MA, USA) was used and reactions were supplemented with 5% DMSO to improve the amplification of yeast DNA [64, 65]. The amplification library was built and used for single-molecule real-time (SMRT) sequencing (Pac Bio, Menlo Park, CA, USA).

### Identification of organic acids by HPLC

The organic acid compounds in koumiss were quantified using a high performance liquid chromatography (HPLC) system (Agilent 1100; Agilent, Santa Clara, CA, USA) with a C_18_ SB-Aq chromatographic column (5 μm, 4.6 mm × 250 mm), solution volume ratio (PBS at a concentration of 10 mmol/L, pH 2.0) of 3:97, and UV detection wavelength of 210 nm [66].

### Taste analysis by a taste-sensing system

The koumiss samples were diluted 1:1 with double-distilled water and centrifuged at 1700×*g* at 4 °C for 10 min. The supernatants were analyzed using the SA402B taste-sensing system (Intelligent Sensor Technology, Inc., Kanagawa, Japan). A random sample placed in the first position was used as the control.

### Statistical analysis

The data are presented as means ± standard error (SE). Statistical analyses were performed using R software (version 3.4.4; R Development Core Team, Vienna, Austria). Statistical differences between two groups were assessed using the Wilcoxon rank sum test. Differences were judged to be statistically significant when *p* < 0.05. Spearman’s rank correlation was used to determine the relationships among species, flavor, and organic acids. Graphs were generated using the ‘ggplot2’ R package (Hadley Wickham, V2.2.1) and GraphPad Prism software (version 7.0; GraphPad Software, Inc., San Diego, CA, USA).

## Supplementary information

**Additional file 1: Figure S1.** Radar curves for koumiss taste data. The red refer to Xilinhot-Urban samples, and blue refer to Xilingol-Rural samples.

## Data Availability

The nucleotide sequence data reported in this study have been deposited in the MG-RAST database (accession No.mgp91551). Other data are available from the corresponding author on reasonable request.

## Abbreviations

LAB: lactic acid bacteria
PC: Principal component
SMRT: Single Molecule Real-Time
TCA: Tricarboxylic acid cycle
RDA: Redundancy analysis
UHT: Ultra-high temperature instantaneous sterilization
