# Supplementary figures and images for: Profiling of koumiss microbiota and organic acids and their effects on koumiss taste
Source: BMC Microbiol. 2020 Apr 10;20:85. doi: 10.1186/s12866-020-01773-z (PMC7149844; doi:10.1186/s12866-020-01773-z)

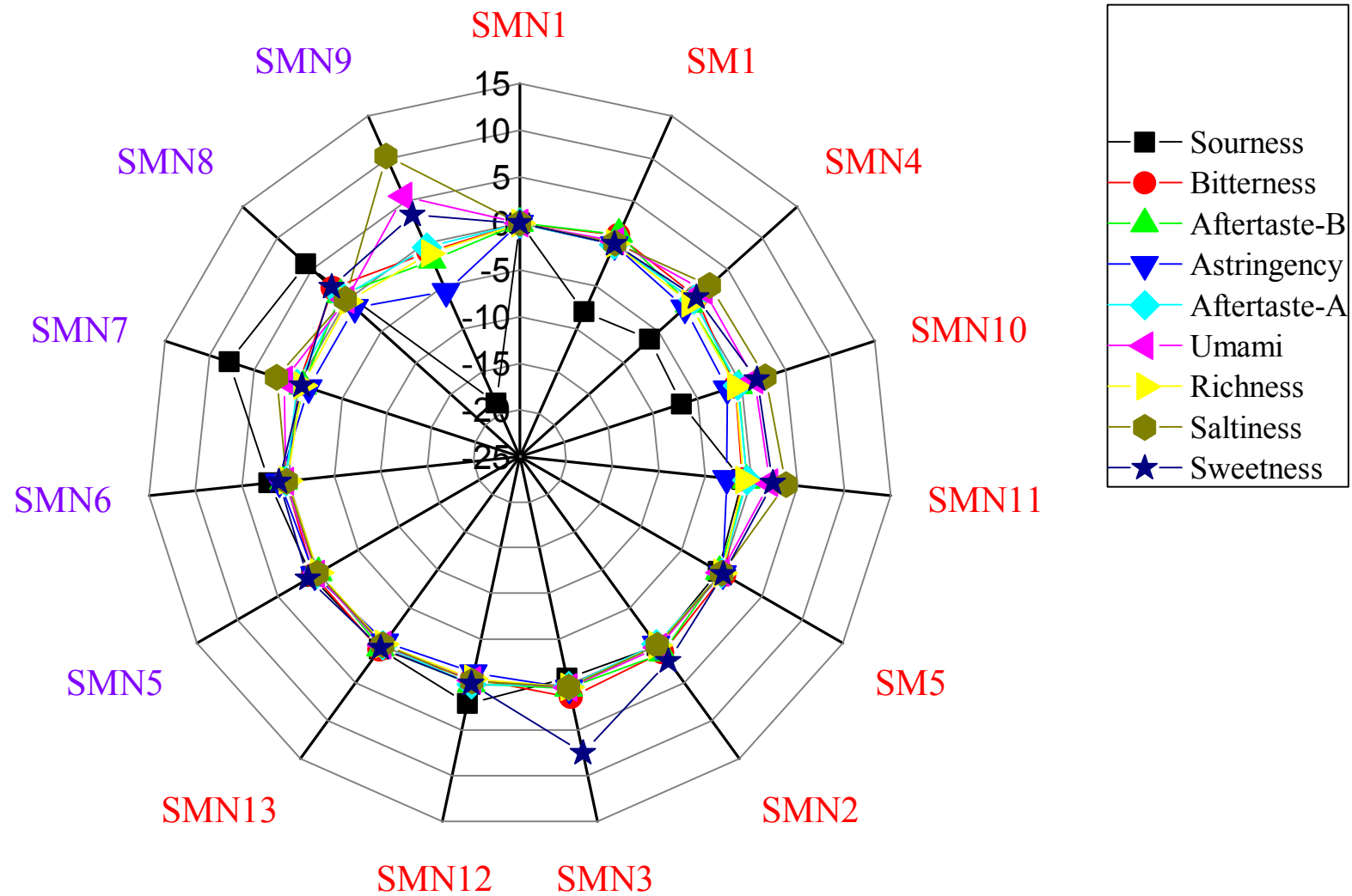

Supplement: Supplementary file 1 — Additional file 1: Figure S1. Radar curves for koumiss taste data. The red refer to Xilinhot-Urban samples, and blue refer to Xilingol-Rural samples. [file 12866_2020_1773_MOESM1_ESM.pdf]
